# Adjusting planting distance plus mineral fertilization to boost growth, yield, and some active ingredients of *Moringa stenopetala*

**DOI:** 10.1038/s41598-026-37068-y

**Published:** 2026-02-12

**Authors:** Ahmed N. Abdelhamid, Mohamed A. Nasser, Laila M. Helmy, Ayman K. Ibrahim, Mahmoud A. A. Ali, Basma T. Abd-Elhalim, Awaad M. Kandeel, Karim M. Hassan

**Affiliations:** 1 Department of Horticulture, Faculty of Agriculture, Ain Shams University, 68-Hadayek Shoubra, Cairo, 11241 Egypt; 2 Department of Agriculture Microbiology, Faculty of Agriculture, Ain Shams University, Hadayek Shoubra, P.O. Box 68, Shubra El-Khaimah, Cairo, 11241 Egypt

**Keywords:** *Moringa stenopetala*, Planting distances, Mineral fertilization, Vegetative growth, Macronutrient content, Flavonoids, L-ascorbic acid, Physiology, Plant sciences

## Abstract

*Moringa stenopetala*, (African Moringa) a drought-resistant and nutritionally rich plant native to East Africa, has gained attention for its potential in food security and medicinal applications. Optimizing agronomic practices such as planting distance and mineral fertilization is crucial to enhancing its growth and phytochemical profile. This study explores how varying planting distances and fertilization levels influence the morphological traits, biomass yield, and nutrient composition of *M. stenopetala*, aiming to identify the most effective combination for maximizing both productivity and nutritional quality. This study investigated the influence of varying planting distance D1 (20 × 60 cm), D2 (40 × 60 cm), D3 (60 × 60 cm) and three mineral fertilization levels F1 (N100 P100 K50 kg/fed), F2 (N200 P200 K100 kg/fed) and F3 (N300 P300 K150 kg/fed) on the growth, yield, and phytochemical composition of *M. stenopetala*. Results indicated that the largest plant height (110.80 cm) was achieved at the highest planting distance (D3), with fertilization level (F3), which also promoted growth parameters. The number of leaves increased by an average of 44.70 with higher fertilization, while the number of branches increased to 14.30 at D3. Biomass yield was maximized with D3F3 238 gm/plant, which also led to the highest nitrogen (2.63%) and phosphorus (0.29%) content in leaves. Micronutrient analysis showed D3F3 produced the highest Fe, Zn, and Mn levels, with Fe reaching 340 ppm and Zn 38.80 ppm. Phytochemical analysis revealed that D3F3 significantly elevated chlorophyll (9.32 mg/100 g), flavonoids (1.50 mg/g), carotenoids (12.92 mg/100 g), tannins (6.50 mg/100 g), and L-ascorbic acid (258 mg/100 g). Overall, the combination of wider planting distance combined with optimal fertilization (D3F3) improved plant growth, nutrient content, and active ingredients, thereby enhancing the nutritional and functional yield plus quality of *M. stenopetala*.

**Supplementary Information:**

The online version contains supplementary material available at 10.1038/s41598-026-37068-y.

## Introduction

Moringa, commonly referred to as the “miracle tree,” is a monogeneric plant belonging to the family Moringaceae. It has been cultivated extensively across tropical regions due to its remarkable nutritional and medicinal properties 1. All parts of the plant—leaves, seeds, pods, bark, and roots—are utilized for various purposes, including food, traditional medicine, and industrial applications. Moringa is recognized among important medicinal plant families^2–7^, and its leaves are particularly rich in essential minerals such as sodium (11.86 ppm), potassium (25.83 ppm), calcium (98.67 ppm), magnesium (107.56 ppm), zinc (148.54 ppm), iron (103.75 ppm), and manganese (13.55 ppm)^8^. Nutritionally, the leaves contain carbohydrate (45.43%), protein (16.15%), fat (9.68%), crude fiber (9.68%), moisture (11.76%), and ash (10.64%), making them a valuable dietary supplement.

The edible leaves are commonly consumed fresh, cooked like spinach, or added to salads and soups. They are known to enhance lactation in nursing mothers and are used to combat malnutrition, especially in infants and postpartum women ^8–11^. In ethnomedicine, *M. oleifera* leaves have been employed to treat a wide range of ailments including skin infections, diarrhea, dysentery, stomach ulcers, and pain. Additionally, moringa has shown potential in regulating blood sugar levels and reducing vascular stress in diabetic patients. Its leaves possess a broad spectrum of pharmacological properties such as antioxidant, diuretic, antihypertensive, anticancer, antipyretic, antiepileptic, anti-inflammatory, antiulcer, and antispasmodic activities^1,12^.

Due to its versatility, every part of the moringa plant is considered highly valuable^13–15^. The leaves retain their nutritional potency when dried into powder, cooked, or consumed fresh. Moringa seeds yield oil that is used in soap production and as cooking oil. The oil’s high oleic acid content also makes it suitable for industrial applications such as fine lubricants and perfumery^16^.

Recent agronomic studies have demonstrated that planting distance and mineral fertilization significantly influence the growth, yield, and phytochemical composition of moringa species. These factors are critical for optimizing the plant’s nutritional and therapeutic potential. Proper spacing enhances resource utilization, biomass accumulation, and leaf yield. For example,^17^ found that plant density directly affects biomass and leaf production in *M. oleifera*, while^18^ showed that both plant population and fertilizer application significantly impact biomass productivity. Mineral fertilization, particularly with NPK, has been shown to elevate phytochemical levels such as chlorophyll, flavonoids, carotenoids, and tannins, which are essential for the plant’s medicinal efficacy. Studies have also indicated that different fertilization regimes can alter the concentration of active compounds in moringa leaves. Notably,^19^ emphasized that nutrient management strategies—including spacing and fertilization—play a pivotal role in determining the phytochemical content, which is vital for both nutritional and medicinal applications.

Studies on planting distance and mineral fertilization are crucial for optimizing the growth, yield, and nutritional quality of Moringa species. These agronomic factors directly influence plant architecture, biomass production, and the concentration of beneficial phytochemicals. Research has shown that appropriate spacing reduces competition and enhances light and nutrient availability, while targeted fertilization—especially with NPK or organic inputs—boosts leaf nutrient content and medicinal properties. Such findings are vital for developing sustainable cultivation practices that maximize Moringa’s potential as a food supplement and therapeutic resource, particularly in regions facing nutritional challenges.

Planting distance and mineral fertilization play a vital role in determining the growth, yield, and quality of moringa plants. Wider spacing generally promotes better air circulation, light penetration, and root development, leading to improved plant height, branching, and leaf production. Likewise, appropriate mineral fertilization—especially with nitrogen, phosphorus, and potassium—enhances biomass yield and boosts the concentration of essential nutrients and phytochemicals. However, excessive fertilization can lead to nutrient imbalances, soil degradation, and environmental pollution. Similarly, overly close planting distances may cause overcrowding, reduced airflow, and increased susceptibility to pests and diseases, ultimately lowering individual plant performance. Therefore, careful management of these factors is essential to maximize the nutritional and medicinal benefits of moringa while maintaining sustainable cultivation practices.

This study hypothesizes that the interaction between planting distance and mineral fertilization levels significantly influences the growth performance, biomass yield, and phytochemical composition of *Moringa stenopetala*. Specifically, it is expected that wider planting distances will reduce intra-species competition, allowing for better light interception, root expansion, and nutrient uptake, while higher levels of mineral fertilization, particularly nitrogen, phosphorus, and potassium—will enhance vegetative growth and increase the concentration of key phytochemicals such as chlorophyll, flavonoids, carotenoids, and L-ascorbic acid. The hypothesis assumes that the optimal combination of these two agronomic factors will not only improve the morphological traits and nutrient content of the plant but also elevate its functional and medicinal value. This approach aims to identify cultivation strategies that maximize both yield and quality, contributing to sustainable agricultural practices and the broader utilization of *M. stenopetala* in nutritional and therapeutic applications.

In light of these hypotheses and findings, the present study aimed to evaluate the effects of planting distance and mineral fertilization on the growth performance, yield, and phytochemical composition of *Moringa stenopetala*. By focusing on cultivation techniques that enhance both nutritional and therapeutic benefits, the research contributes to the development of optimized agronomic practices for this valuable plant species.

## Materials and methods

### Plant samples and treatments

A field experiment was conducted in Wadi El-Natron, Behira Governorate (30°19’46.1"N 30°15’01.6"E), Egypt from March to November, to evaluate the effects of planting distance and mineral NPK fertilization on the growth, yield, and chemical composition of *Moringa stenopetala* (African Moringa).

Seeds were sourced from the National Research Center, Giza, Egypt and sown directly in sandy soil under a drip irrigation system with water salinity of 400 ppm.

- The analysis showed that the soil classified as sandy texture,
- pH of (8.76), Ec (1.02 dS m^–^1).
- Climate condition: The average of temperature (23 °C to 38 °C) for maximum temperature, (14 °C to 24 °C) for minimum temperature during the growing period and the average Relative Humidity was 35 to 65%.

**Experimental Design**:

- **Split-plot design** with 4 replications.
- **Main plots**: Three planting distances:
  - D1 20 × 60 cm (35,000 plants/fed).
  - D2 40 × 60 cm (17,500 plants/fed).
  - D3 60 × 60 cm (11,660 plants/fed).
- **sub-plot**: Three NPK fertilizer levels:
  - F1 N100 P100 K50 kg/fed.
  - F2 N200 P200 K100 kg/fed.
  - F3 N300 P300 K150 kg/fed.
- **Total plants**: 360 (9 treatments × 4 reps × 10 plants each).

**Fertilizer Sources**:

- **Nitrogen**: Ammonium nitrate (33.5%).
- **Phosphorus**: Mono ammonium phosphate (MAP, N12 P61 K0).
- **Potassium**: Solo-potash (50%).

Fertilizers were applied in equal portions during May, July, September, and October.

**Data Collection**:

- **Growth parameters**: Plant height and number of lateral branches.
- **Yield**: Fresh and dry weight per plant and per feddan, measured over five harvests.
- **Chemical analysis**:
  - **Nitrogen**: Pregl (1945) method^20^.
  - **Phosphorus**: Jackson (1967) method^21^.
  - **Potassium**: Brown and Lilleland (1946) method^22^.
- NPK content expressed as g/100 g dry leaf weight.

**Determination of active ingredients.**

**Total Carotenoids**:

Measured by a colorimetric method (A.O.A.C., 1990)^23^ and expressed as mg/100 g fresh weight.

**Total Flavonoids**:

Determined via a colorimetric assay involving methanolic extraction, NaNO₂, AlCl₃, and NaOH. Absorbance was read at 510 nm, and results were expressed as mg catechin/g fresh weight^24^.

**Total Tannins**:

Assessed using the Folin and Denis method^25^ with absorbance at 760 nm. Concentration was calculated using a pyrogallol standard and expressed as mg/100 g dry weight.

**L-Ascorbic Acid (Vitamin C)**:

Estimated by titration with 2,6-dichlorophenolindophenol dye after extraction in 3% oxalic acid, and expressed as mg/100 g fresh weight^26^.

### Statistical analysis

The analysis of variance (ANOVA) approach was used for all statistical analyses of the various features. Duncan’s multiple range tests were used to examine data for statistically significant differences. According to Snedecor and Cochran (1982)^27^. All statistical conclusions were drawn at a significance level of *P* < 0.05. The SAS package’s analysis of variance was used to statistically examine the data. The heatmap correlation was used to detect the relationship between the parameters by the R Studio program and the “metan” package in the R software environment. The RStudio program with “tidyverse” package in the R software environment was used to create a heatmap, which offered insightful information.

## Results

### Vegetative growth parameters

**Fig. 1:**
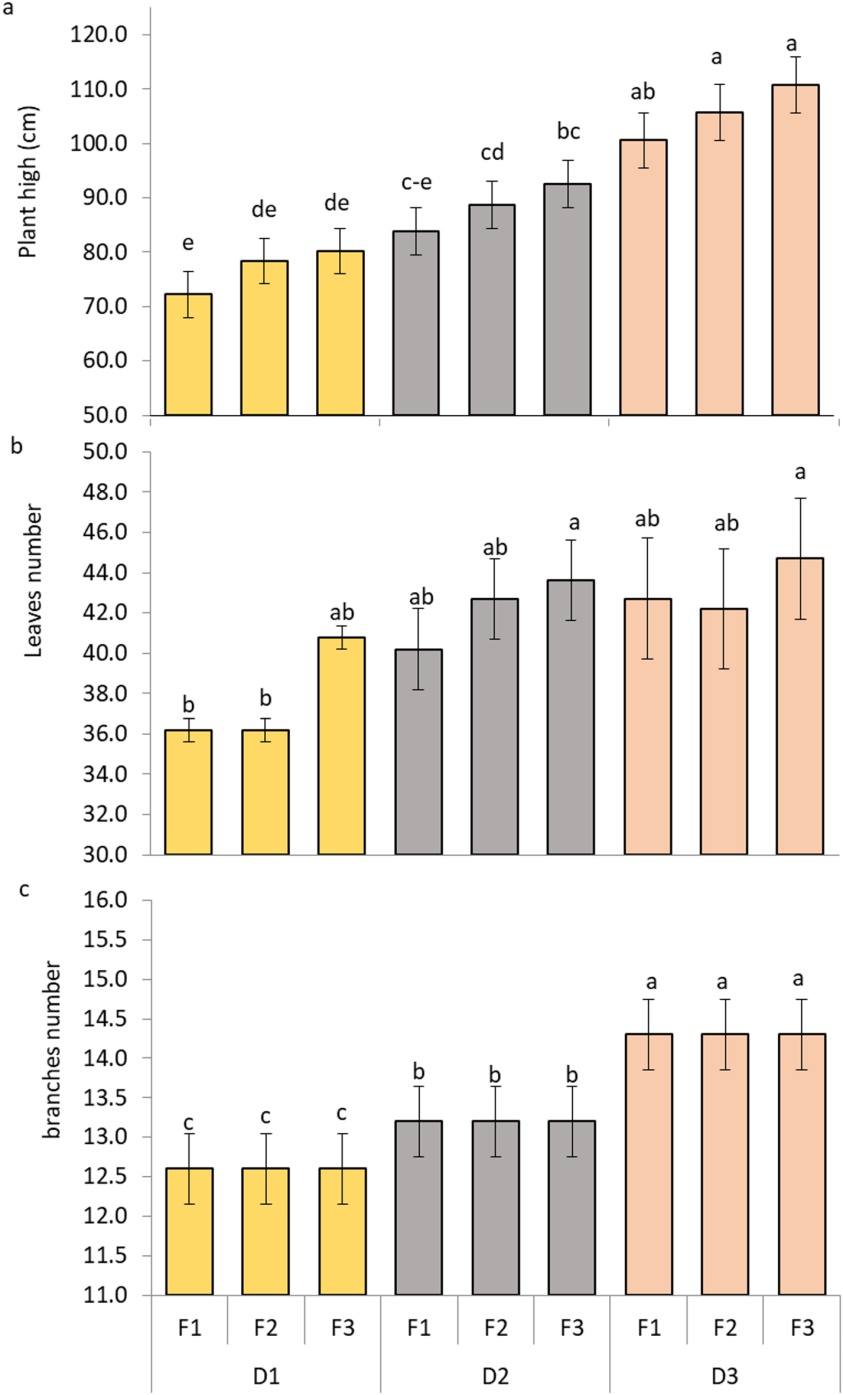
Impact of NPK fertilizer and planting distance on vegetative growth of *M. stenopetala* plants (**a**) plant height (**b**) leaves number (**c**) branches number. The Tukey test indicates that there is a significant difference at *p* ≤ 0.05 between means that do not share the letters for each variable in each bar.

Planting distances and fertilization treatments affected the vegetative growth of plants, such as plant height, number of leaves, and number of branches (Fig. 1).

In Fig. 1, Plant height was pointedly altered via planting distance (D) and fertilizer level (F) (*p* < 0.05). Hence, plant height gradually increased with wider plant distance and higher fertilizer amount. More, the highest plants were documented with the D3F3 and D3F2 treatments, reaching around 110.80 and 105.70 cm, in that order with same statistical letter a. On the other hand, shortest moringa plants were noted in the D1F1 treatment (roughly 72.20 cm letter e), which got the lowest fertilizer amount and the precise planting distance.

Leaves number per plant was substantially influenced by D and F (*p* < 0.05). The greatest leaf numbers were found under D3F3 and D2F3 treatments that have around 45 leaves per plant with same statistical letter a. On the other hand, the minimal leaf numbers detected under D1F1 and D1F2, with an average nearly 35 leaves per plant, and were assigned letter b.

Likewise, branch number was considerably affected by both planting distance and fertilizer rate. In other words, maximum branches number were noted under the wider distance treatments (D3F1, D3F2, and D3F3), with an average of about 14.30 branches per plant, all of which were assigned to the same statistical group (letter a) according to Duncan’s multiple range test. In contrast, the narrowest spacing treatments (D1F1, D1F2, and D1F3) recorded the lowest branch number, averaging approximately 12.60 branches per plant with letter c.

Inclusive, the influences of wider plant distance and higher fertilizer amount substantially enhanced vegetative growth traits, proving the interactive of these two agronomical features in boosting plant vigor.

### Yield

**Fig. 2:**
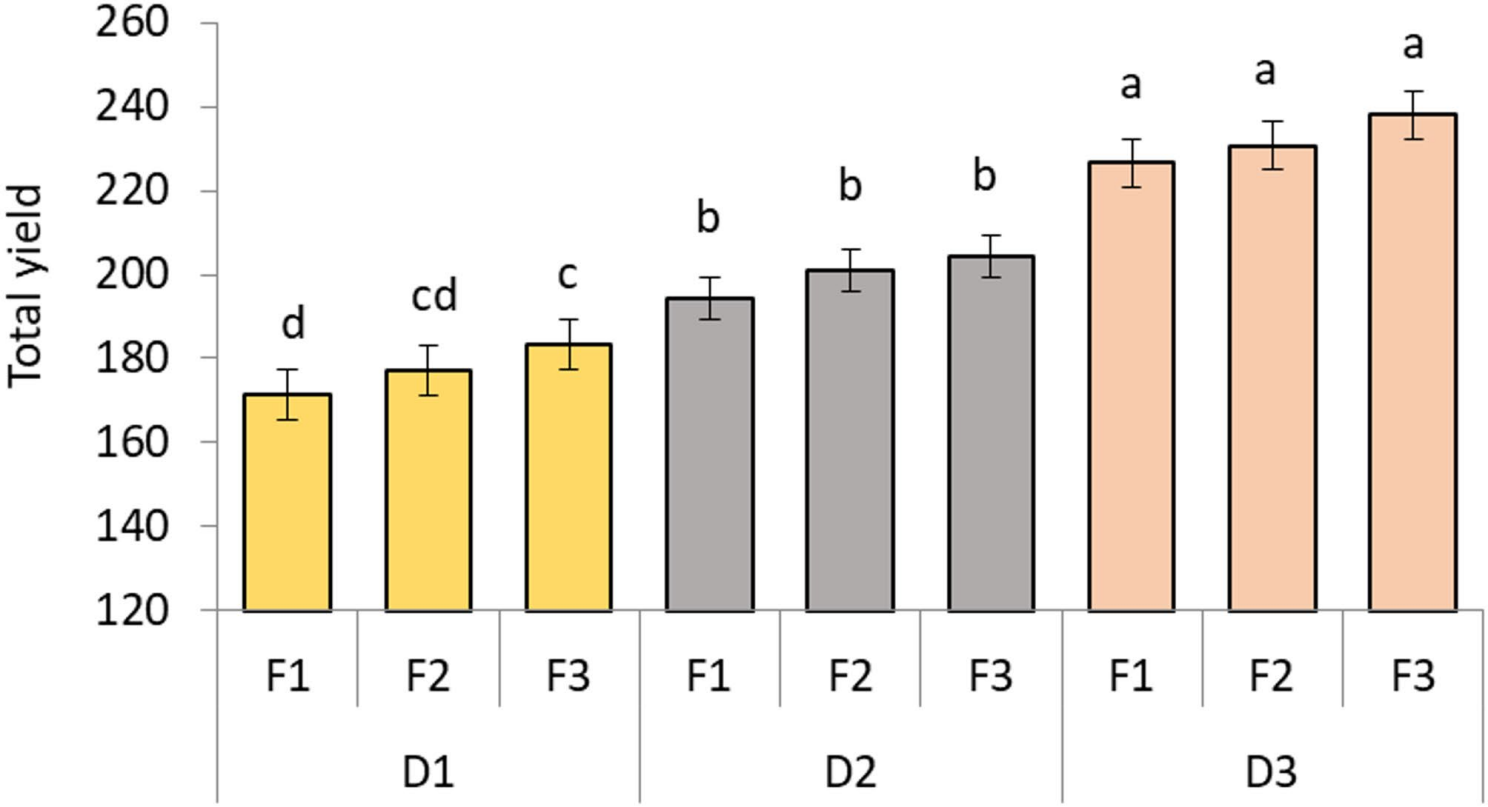
Effect of planting distance and fertilization on yield of *M. stenopetala*. The Tukey test indicates that there is a significant difference at *p* ≤ 0.05 between means that do not share the letters for each variable in each bar.

Total yield was meaningfully influenced by D and F (*p* < 0.05). The maximum total yields are noted in the wider distance D3F1, D3F2, and D3F3 treatments, varying between 226.60, 230.70 and 238 gm per plant, and appointed to the same statistical group letter a. In contrast, the lowest total yield was obtained from the D1F1 treatment, which yielded roughly 171.50 gm per plant, and was marked letter d. Wider distance, combined with superior fertilizer rate, improves yield, possible regarding enhanced light capture, mineral uptake, and decreased competition between plants (Fig. 2).

### Macronutrient content

**Fig. 3:**
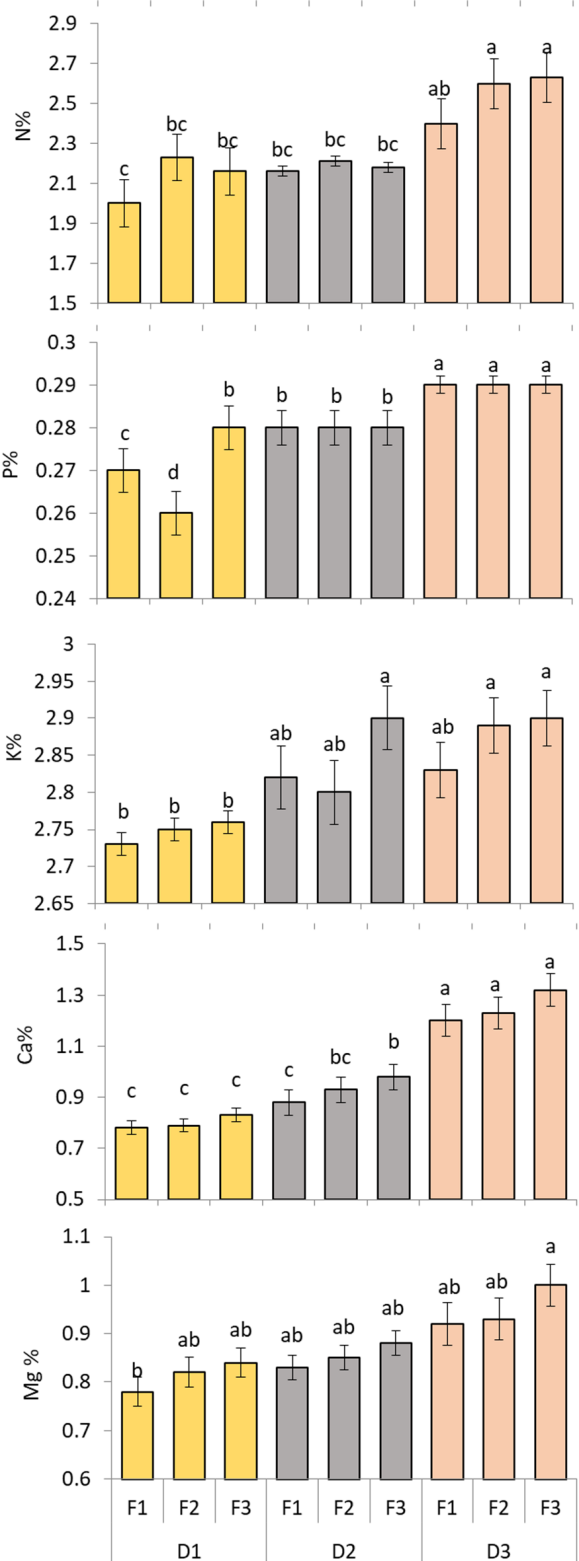
Effect of planting distance and NPK fertilization on macronutrient content of *M. stenopetala* leaves. The Tukey test indicates that there is a significant difference at *p* ≤ 0.05 between means that do not share the letters for each variable in each bar.

The analysis of variance (ANOVA) showed that D and F substantially affected the macronutrient levels of the plants (*p* < 0.05) (Fig. 3). Nitrogen level heightened significantly with wider distance and higher fertilizer amount. The maximum N level was noted in D3F2 and D3F3 (something like 2.6%), both named the same statistical letter a, while the lowest value was observed in D1F1 (around 2.0%, letter c).

Phosphorus concentration also altered substantially between treatments. The top P content was found in D3F1, D3F2, and D3F3, around 0.29% (letter a). The lowest P value was recorded in D1F2 (nearby 0.26%, letter d), representing that greater fertilizer amount and wider distance enhance P uptake effectiveness.

Potassium values were radically superior in treatments of D2F3, D3F2, and D3F3 (nearly 2.90%, letter a) compared among D1F2, which demonstrated the minimal K values (approximately 2.73%, letter b). These findings indicate that improved fertilizer rate combined with wider distance boosts K concentration in plants.

Calcium concentration confirmed a comparable tendency, with the maximum levels detected in D3F1, D3F2, and D3F3 treatments surrounding 1.2% with letter a. The minimal Ca values were detected in D1F1, D1F2, and D1F3 treatments roughly 0.8% with letter c. In fact, this indicates that Ca uptake is improved under wider distance and adequate fertilization.

Magnesium achieved maximum levels in D3F3 treatment approximately 1.0% with letter a, while the minimal level took place in D1F1 nearby 0.8% with letter b. The results point out that wider distance and higher fertilizer availability affect Mg uptake in plants.

To illustrate, the data establishes that wider planting distance joint with higher fertilizer rates meaningfully progresses the macronutrients uptake and accumulation in moringa leaves.

### Micronutrient content

**Fig. 4:**
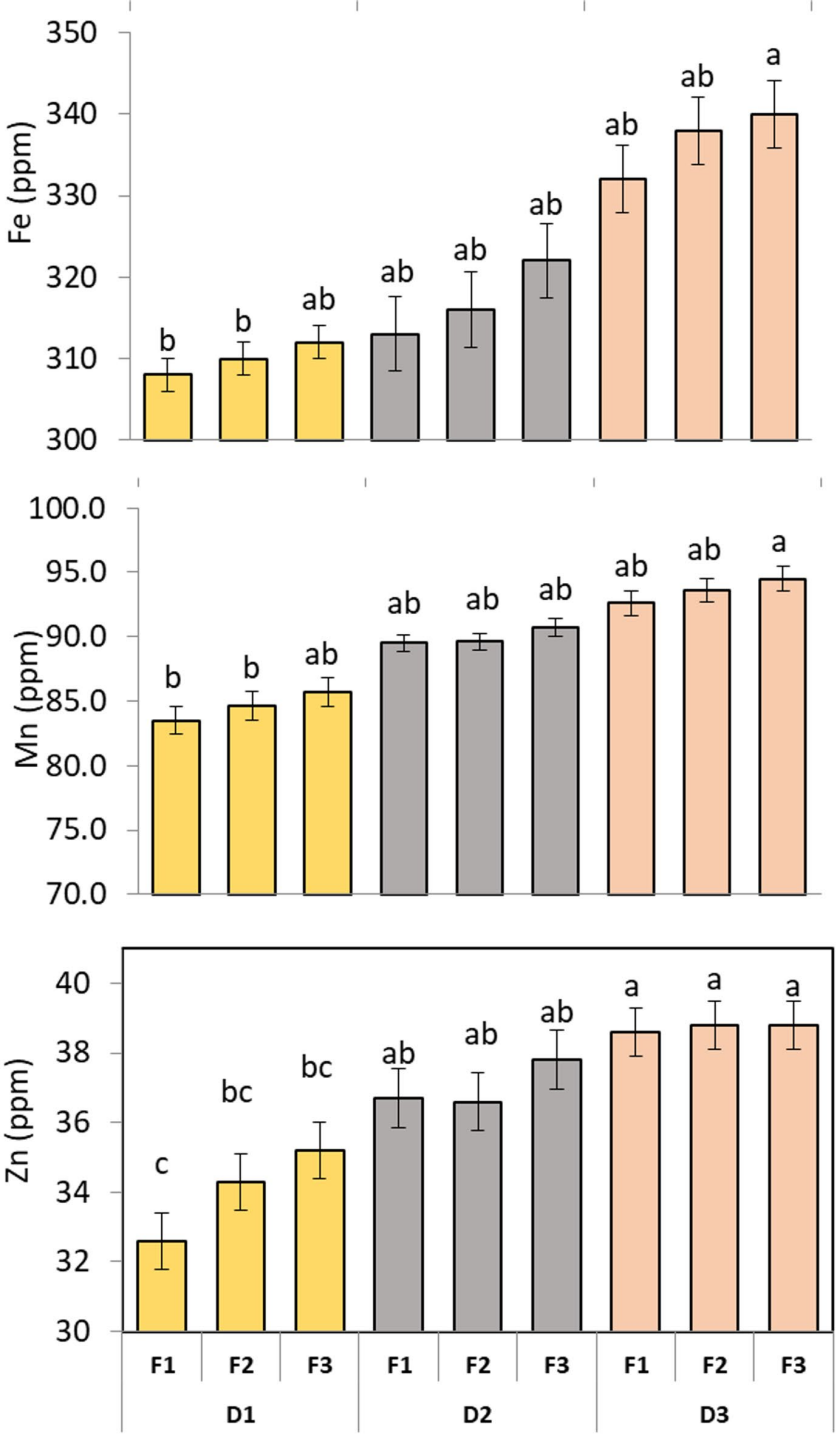
Effect of planting distance and NPK fertilization on micronutrient content of *M. stenopetala* leaves. The Tukey test indicates that there is a significant difference at *p* ≤ 0.05 between means that do not share the letters for each variable in each bar.

As shown in (Fig. 4), ANOVA analysis showed that D and F noticeably altered the micronutrient levels of moringa leaves (*p* < 0.05). Iron accumulation improved significantly with wider planting distance and higher fertilizer amounts. To declare, the maximum Fe level was noted in D3F1, D3F2 and D3F3 something like 340 ppm with same statistical letter a, while the lowest value was observed in D1F1 (near 308 ppm, letter c). Likely, Manganese showed higher levels under D3 and all F amounts 94.50 ppm, while decreased content under D1 with all fertilizer rates 83.50 ppm. In the same manner, Zinc recorded the lowest values around 32.6 ppm with letter c underD1F1 treatment. On the other hand, Zn registered higher content under D3 combined with different fertilizer rates nearby 38.80 ppm with letter a.

To conclude, the results showed that larger planting distance joint with higher fertilizer amounts expressively improves the macronutrients uptake and accumulation in moringa.

### Active ingredients

**Fig. 5:**
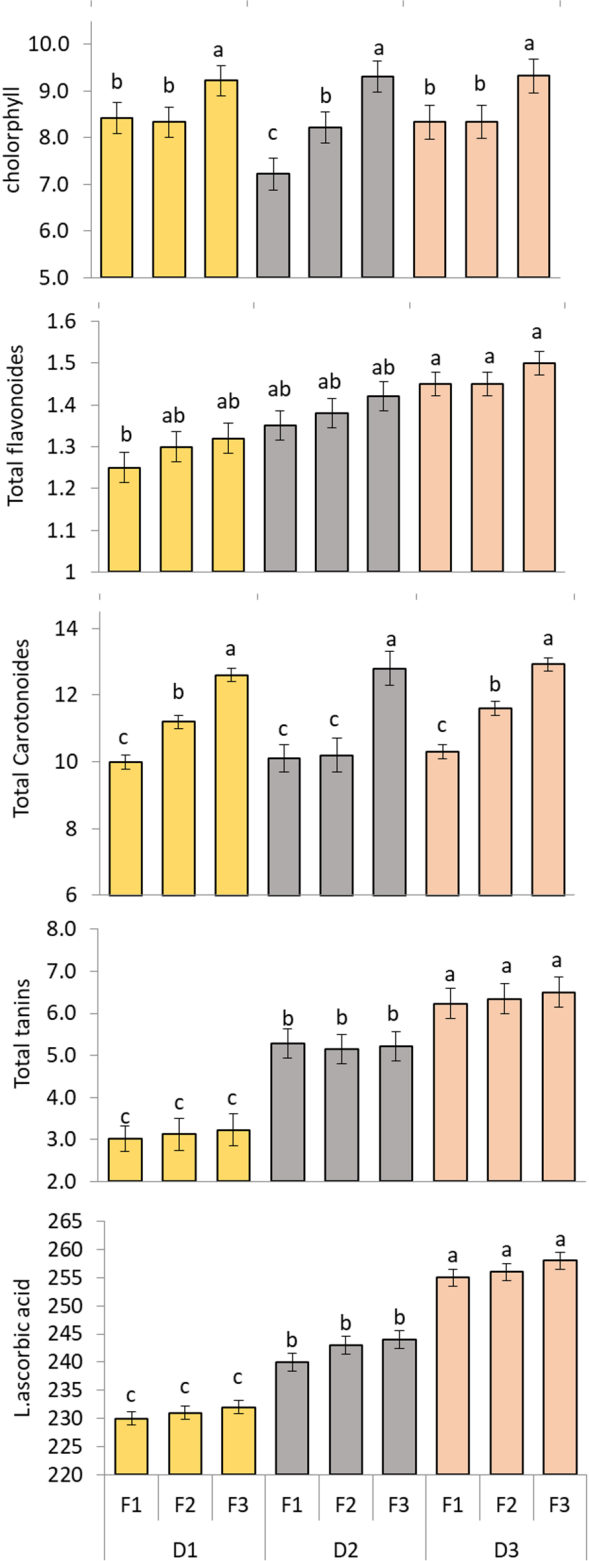
Effect of planting distance and NPK fertilization on active ingredients (Chlorophyll (mg/100 g fresh weight), Total Flavonoid (mg/g fresh weight), Total Carotenoid (mg/100 g fresh weight), Total Tannins (mg/100 g dry weight) and L-Ascorbic Acid) (mg/100 g fresh weight) of *M. stenopetala* leaves. The Tukey test indicates that there is a significant difference at *p* ≤ 0.05 between means that do not share the letters for each variable in each bar.

Data in Fig. 5 show the effect of planting distance and NPK fertilization on active ingredients of *M. stenopetala* leaves. The treatments affect Phytochrome such as chlorophyll, total flavonoids and total carotenoids. The chlorophyll content of leaves increased with F3 treatment with all planting distances (9.22, 9.31 and 9.32 mg/100 g respectively). On the other hand, D3 increased the total flavonoids without any significant effect of fertilization levels (1.45, 1.45 and 1.50 mg/g respectively). Total carotenoids were not affected by planting distance while affected by fertilization levels. The highest values of total carotenoids were obtained with D1F3, D2F3 and D3F3 (12.60, 12.80 and 12.92 mg/100 g respectively). Also, the total tannins were affected by planting distance. The D3 obtained highest values of total tannins by (6.50 mg/100 g) compared to D1(3.02 mg/100 g). While the total tannin content was not affected by the fertilization rate. Concerning L-ascorbic acid (mg/100 g fresh weight) the highest values were obtained with D3F1, D3F2 and D3F3 (255.00, 256.00 and 258.00 respectively). Generally, the highest values of active ingredients were obtained with D3F3.

**Fig. 6:**
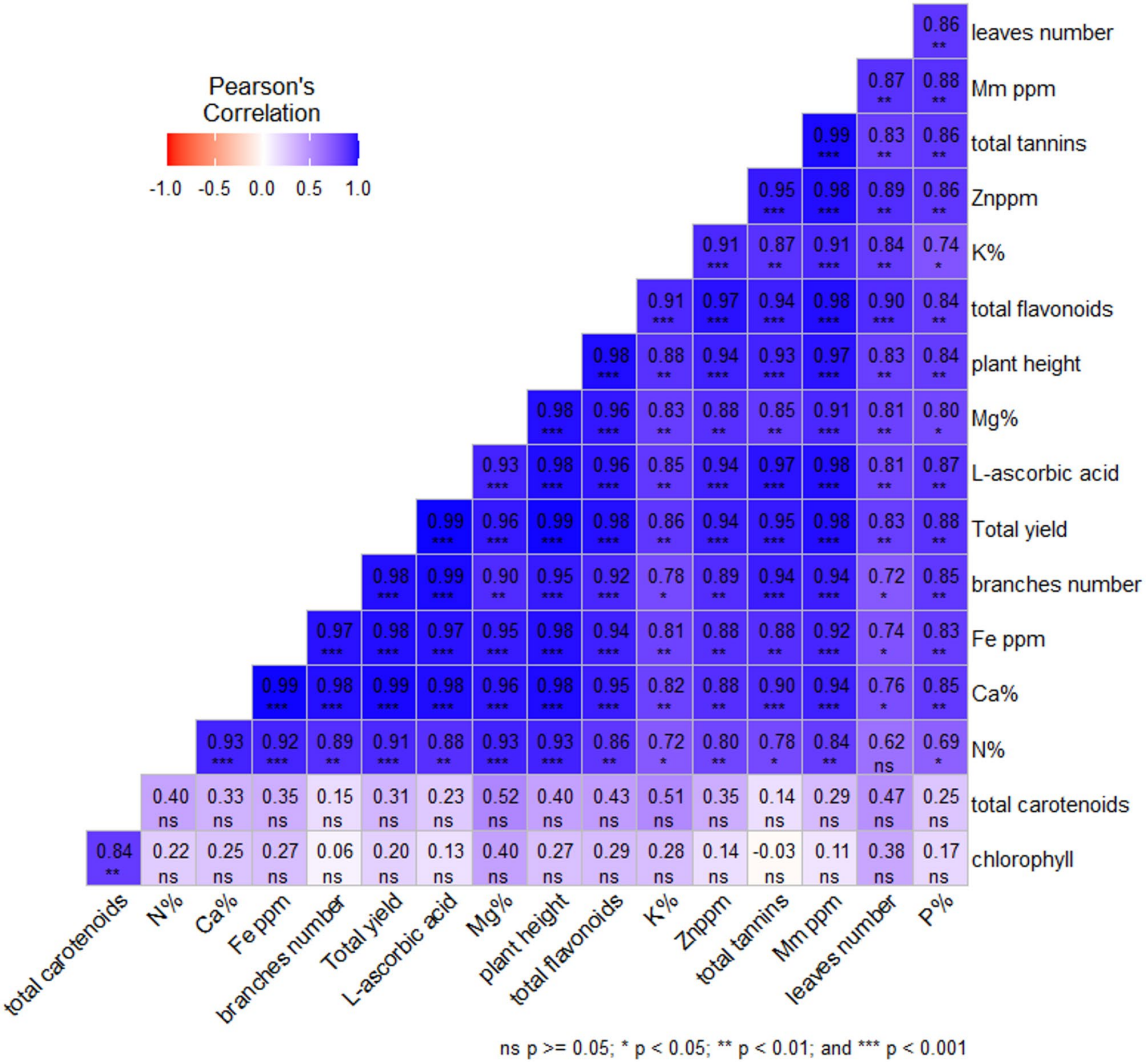
Heat map correlation coefficients between different pairs of *M. stenopetala* characteristics estimated under different planting distance and NPK fertilization. *, ** and ***: correlation is significant at 0.05, 0.01 and 0.001 level of significance.

The study estimated correlation coefficients between different pairs of *M. stenopetala* characteristics under various planting distances and NPK fertilization, which are expressed in heat maps (Fig. 6). The calculated correlation coefficients, except for chlorophyll and total carotenoids, exhibited significant and positive associations between all pairs.

**Fig. 7:**
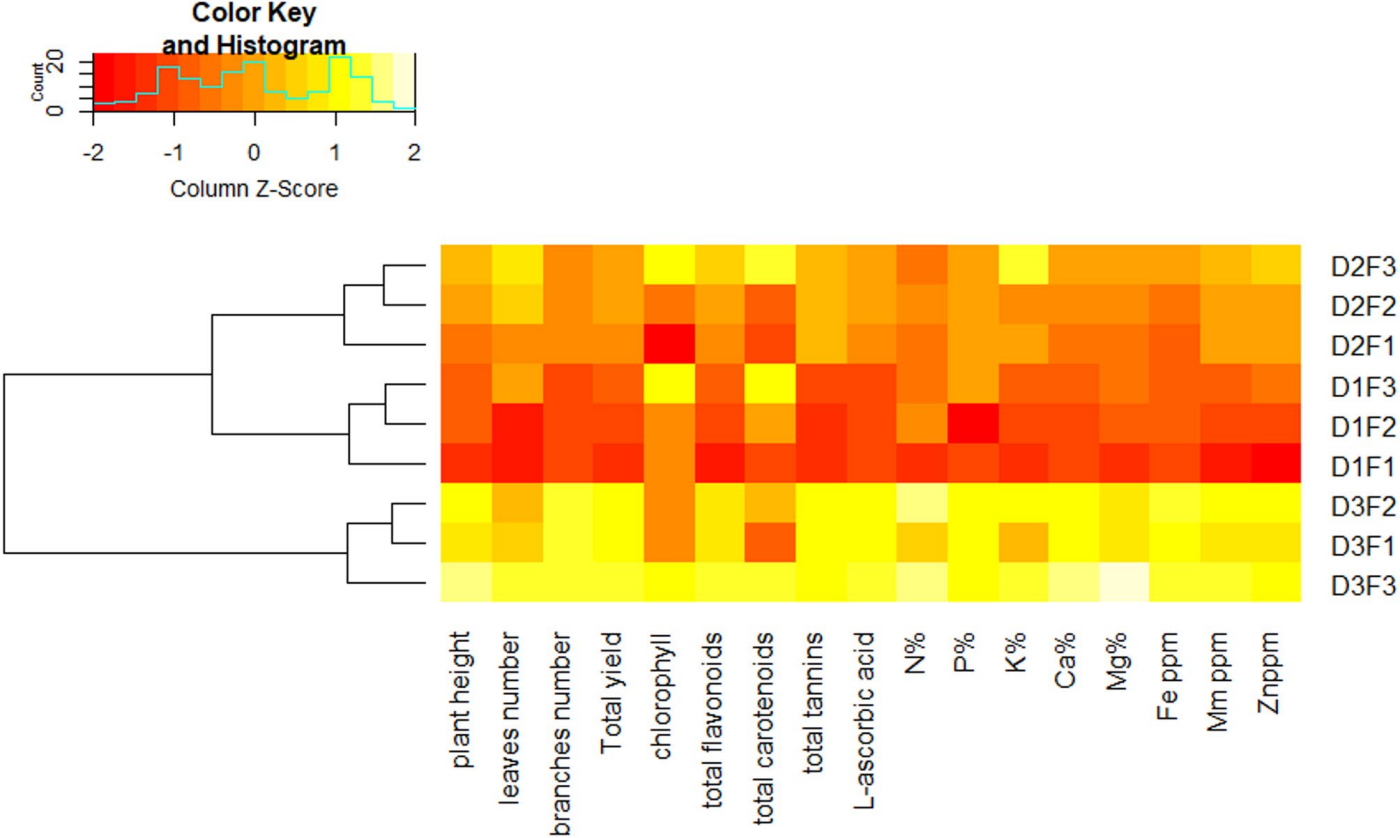
Heatmap for the evaluated parameters of *M. stenopetala* grown at different planting distances and NPK fertilization. Parameters in Yellow color and red color reveal high and low values for the corresponding parameters, respectively.

Clustered heatmap: The clustered heatmap provides a clear visual demonstration that the different planting distances and different levels of NPK fertilization significantly affected the growth and biochemical and nutrient contents of *M. stenopetala* plants; parameters in yellow color and red color reveal high and low values for the corresponding parameters, respectively (Fig. 7). Growth parameters (plant height, number of leaves, number of branches, and total yield), nutrients (N, P, K, Ca, Mg, Fe, Mn, and Zn), and active ingredients (chlorophyll, total flavonoids, total carotenoids, total tannins, and L-ascorbic acid) exhibit a pronounced increase, especially with distance at 60 × 60 cm (D3), along with the highest fertilization level, F3 (N300 P300 K150 kg/fed).

## Discussion

### Vegetative growth performance

The findings of this study highlight the significant influence of planting distance and mineral fertilization on the vegetative growth of *Moringa stenopetala*. Wider spacing (D3) combined with higher fertilization levels (F3) resulted in the tallest plants, with a 34.84% increase in height compared to the control. This improvement is attributed to reduced competition for light, water, and nutrients, allowing for better root expansion and canopy development. The number of leaves and branches also increased under these conditions, indicating enhanced photosynthetic capacity and structural growth. These results align with previous studies on crops like millet and okra, where wider spacing facilitated better resource allocation and vegetative development^17,28^.

### Yield enhancement

Yield analysis revealed that both planting distance and fertilization significantly affected biomass production. The highest total yield of 120 g per plant was recorded under the D3F3 treatment, demonstrating a synergistic effect between optimal spacing and nutrient supplementation. Wider spacing allowed for more extensive vegetative growth, while higher NPK fertilization improved nutrient availability, supporting vigorous development. These findings confirm that strategic agronomic practices can substantially increase productivity in *M. stenopetala*, consistent with similar results in *M. oleifera* and other leafy crops^22,29^.

### Macronutrient accumulation

Macronutrient content in the leaves was positively influenced by planting distance and fertilization. Nitrogen levels increased under D3F3, enhancing protein synthesis and vegetative vigor. Phosphorus content was highest under D3, though fertilization had limited impact at wider spacings. Potassium levels peaked under D2F3, D3F2, and D3F3, supporting water regulation and stress tolerance. Calcium content was significantly higher under D3, over D1, while Magnesium was most abundant in D3F3^30,32^. These results suggest that wider spacing improves nutrient uptake, and fertilization further enhances macronutrient accumulation.

### Micronutrient content

Micronutrient analysis showed that iron and zinc concentrations were highest under D3, indicating that wider spacing facilitates better uptake of these essential elements. Fertilization levels did not significantly alter Fe and Zn under D3, suggesting spacing plays a more dominant role. Manganese content, however, was highest under D3F3 compared to D1F1, reflecting the combined effect of spacing and fertilization on micronutrient absorption. These findings support earlier research showing that optimal planting arrangements improve micronutrient accumulation in leafy vegetables^33–37^.

### Phytochemical composition

The phytochemical profile of *M. stenopetala* leaves was significantly enhanced by the D3F3 treatment. Chlorophyll content reached 9.32 mg/100 g, indicating improved photosynthetic efficiency. Flavonoids and carotenoids, known for their antioxidant properties, also peaked under D3F3, suggesting that optimal spacing and fertilization promote secondary metabolite synthesis. Tannin levels varied across treatments, with the highest values observed in D3, D1, and D2, implying that spacing may influence phenolic compound production through stress modulation. L-ascorbic acid (Vitamin C) content was also elevated under D3F3, reinforcing the nutritional benefits of this treatment. These results are consistent with previous studies on moringa species, which found that nutrient management positively affects phytochemical accumulation^31,32^.

### Conclusion and implications

Overall, the study demonstrates that the combination of wider planting distance (D3) and higher NPK fertilization (F3) significantly improves vegetative growth, yield, macronutrient and micronutrient content, and phytochemical composition in *M. stenopetala*. These findings validate the importance of strategic agronomic practices in maximizing crop productivity and quality. While the D3F3 treatment proved optimal in this study, future research should explore the long-term environmental sustainability and economic feasibility of these practices. Additionally, caution should be taken to avoid excessive fertilization, which may lead to environmental concerns or diminishing returns^32,33^. The results contribute valuable insights for improving moringa cultivation, particularly in regions where it serves as a key nutritional and medicinal resource.

## Conclusion

A systematic research of the effects of planting distance and NPK fertilizer on *Moringa stenopetala* yielded numerous major discoveries with practical implications. The study found that optimizing planting distance, especially at 60 × 60 cm (D3), along with the highest fertilization level F3 (N300 P300 K150 kg/fed) of nitrogen, phosphorus, and potassium, significantly improved vegetative growth, biochemical composition, micronutrient content, and overall yield. The observed gains in plant height, leaf number, and branching with wider spacing were attributed to increased resource availability and reduced competition, which facilitated more vigorous plant development.

Furthermore, the increased amounts of active compounds such as chlorophyll, flavonoids, carotenoids, and L-ascorbic acid under optimal fertilization demonstrate the relevance of balanced nutrient supply in improving not just yield but also the nutritional and medicinal quality of M. stenopetala leaves. Higher micronutrient concentrations (Fe, Zn, and Mn) linked with wider spacing and higher fertilization enhance the plant’s value as a nutrient-dense crop.

These findings support and build on prior research on Moringa, demonstrating that targeted agronomic techniques can significantly increase crop output and quality. However, the quest of maximal yield and phytochemical enhancement must take into account environmental sustainability, as excessive fertilization can cause nutrient runoff and ecological imbalance. Future study should concentrate on sustainable fertilization strategies, economic viability, and the long-term environmental consequences of these approaches.

**Advantages of the Study**:

- Provides a clear agronomic framework for enhancing M. stenopetala productivity and nutritional quality.
- Offers practical recommendations for farmers and researchers on optimal spacing and fertilization.
- Contributes to food security and health promotion through improved cultivation of a nutrient-rich crop.

**Disadvantages of the Study**:

- Limited assessment of long-term environmental impacts of high fertilization rates.
- Economic analysis of input costs versus yield benefits was not included.
- The study was conducted under specific agroecological conditions, which may limit generalizability to other regions.

### Future prospects

Future prospects for the cultivation and utilization of *M. stenopetala* encompass several promising avenues to maximize its potential benefits:

- Firstly, further research should explore the integration of sustainable and environmentally friendly fertilization practices, such as organic amendments or biofertilizers, to mitigate environmental impacts while maintaining high yields and nutrient quality. This would support sustainable agriculture and promote eco-friendly cultivation techniques.
- Secondly, breeding and genetic improvement programs could be initiated to develop higher-yielding, pest-resistant, and climate-adapted moringa varieties. Such innovations would enhance productivity and resilience, especially in regions facing climate variability.
- Thirdly, expanding research on the phytochemical profile and medicinal properties related to different cultivation practices could lead to targeted utilization of moringa in pharmaceuticals, nutraceuticals, and functional foods. Detailed studies on the bioavailability and pharmacological activities of active compounds are essential for developing health-related products.
- Additionally, exploring the post-harvest processing, preservation, and value addition techniques can improve the shelf life and marketability of moringa leaves and other products, fostering local entrepreneurship and increasing income for farmers.

Finally, integrating moringa cultivation into agroforestry systems and sustainable land management practices can enhance biodiversity, soil health, and ecological balance. Promoting farmer education and extension services will be vital to adopting best practices and maximize the benefits of the crop. Overall, the future of *Moringa stenopetala* research should aim at sustainable intensification, product diversification, and scaling up agro-industrial processing, thereby unlocking its full potential as a nutritionally rich, medicinal, and economically valuable crop.

## Supplementary Information

Below is the link to the electronic supplementary material.

Supplementary Material 1

## Data Availability

Data sets generated during the current study are available from the corresponding author upon reasonable request.
