# Supplementary material for: Adjusting planting distance plus mineral fertilization to boost growth, yield, and some active ingredients of Moringa stenopetala
Source: Sci Rep. 2026 Feb 12;16:6353. doi: 10.1038/s41598-026-37068-y (PMC12905418; doi:10.1038/s41598-026-37068-y)
Supplement: Supplementary file 1 — Supplementary Material 1 [file 41598_2026_37068_MOESM1_ESM.docx]

Table S1. Impact of NPK fertilizer and planting distance on vegetative growth of *M. stenopetala* plants.

| treatments | plant height (cm) | leaves number | branches number |
| --- | --- | --- | --- |
| D1F1 | 72.20 ± 4.18 ^e^ | 36.20 ± 2.66 ^b^ | 12.60 ± 0.45 ^c^ |
| D1F2 | 78.30 ± 4.18 ^de^ | 36.20 ± 2.66 ^b^ | 12.60 ± 0.45 ^c^ |
| D1F3 | 80.20 ± 4.18 ^de^ | 40.80 ± 2.66 ^ab^ | 12.60 ± 0.45 ^c^ |
| D2F1 | 83.90 ± 4.36 ^c-e^ | 40.20 ± 1.76 ^ab^ | 13.20 ± 0.45 ^b^ |
| D2F2 | 88.70 ± 4.36 ^cd^ | 42.70 ± 1.76 ^ab^ | 13.20 ± 0.45 ^b^ |
| D2F3 | 92.60 ± 4.36 ^bc^ | 43.60 ± 1.76 ^a^ | 13.20 ± 0.45 ^b^ |
| D3F1 | 100.60 ± 5.10 ^ab^ | 42.70 ± 1.32 ^ab^ | 14.30 ± 0.45 ^a^ |
| D3F2 | 105.70 ± 5.10 ^a^ | 42.20 ± 1.32 ^ab^ | 14.30 ± 0.45 ^a^ |
| D3F3 | 110.80 ± 5.10 ^a^ | 44.70 ± 1.32 ^a^ | 14.30 ± 0.45 ^a^ |

* The Tukey test indicates that there is a significant difference at p≤ 0.05 between means that do not share the letters for each variable in each column.

** planted at a distance: D1= 20 × 60 cm, D2= 40 × 60 cm, D3= 60 × 60 cm.

*** fertilized with NPK fertilizer: F1= N100 P100 K50, F2= N200 P200 K100, F3= N300 P300 K150.

Table S2. Effect of planting distance and fertilization on yield of *M. stenopetala*.

| treatments | total yield (g) |
| --- | --- |
| D1F1 | 171.50 ± 5.90 d |
| D1F2 | 177.10 ± 5.90 cd |
| D1F3 | 183.30 ± 5.90 c |
| D2F1 | 194.40 ± 5.07 b |
| D2F2 | 200.90 ± 5.07 b |
| D2F3 | 204.40 ± 5.07 b |
| D3F1 | 226.60 ± 5.77 a |
| D3F2 | 230.70 ± 5.77 a |
| D3F3 | 238.00 ± 5.77 a |

* The Tukey test indicates that there is a significant difference at p≤ 0.05 between means that do not share the letters for each variable in each column.

** planted at a distance: D1= 20 × 60 cm, D2= 40 × 60 cm, D3= 60 × 60 cm.

*** fertilized with NPK fertilizer: F1= N100 P100 K50, F2= N200 P200 K100, F3= N300 P300 K150.

Table S3. Effect of planting distance and NPK fertilization on macronutrient content of *M. stenopetala* leaves.

| treatments | N% | P% | K% | Ca% | Mg% |
| --- | --- | --- | --- | --- | --- |
| D1F1 | 2.00 ± 0.12 c | 0.27 ± 0.005 c | 2.73 ± 0.015 b | 0.78 ± 0.03 c | 0.78 ± 0.03 b |
| D1F2 | 2.23 ± 0.12 bc | 0.26 ± 0.005 d | 2.75 ± 0.015 b | 0.79 ± 0.03 c | 0.82 ± 0.03 ab |
| D1F3 | 2.16 ± 0.12 bc | 0.28 ± 0.005 b | 2.76 ± 0.015 b | 0.83 ± 0.03 c | 0.84 ± 0.03 ab |
| D2F1 | 2.16 ± 0.03 bc | 0.28 ± 0.004 b | 2.82 ± 0.043 ab | 0.88 ± 0.05 c | 0.83 ± 0.03 ab |
| D2F2 | 2.21 ± 0.03 bc | 0.28 ± 0.004 b | 2.80 ± 0.043 ab | 0.93 ± 0.05 bc | 0.85 ± 0.03 ab |
| D2F3 | 2.18 ± 0.03 bc | 0.28 ± 0.004 b | 2.90 ± 0.043 a | 0.98 ± 0.05 b | 0.88 ± 0.03 ab |
| D3F1 | 2.40 ± 0.13 ab | 0.29 ± 0.002 a | 2.83 ± 0.038 ab | 1.20 ± 0.06 a | 0.92 ± 0.04 ab |
| D3F2 | 2.60 ± 0.13 a | 0.29 ± 0.002 a | 2.89 ± 0.038 a | 1.23 ± 0.06 a | 0.93 ± 0.04 ab |
| D3F3 | 2.63 ± 0.13 a | 0.29 ± 0.002 a | 2.90 ± 0.038 a | 1.32 ± 0.06 a | 1.00 ± 0.04 a |

* The Tukey test indicates that there is a significant difference at p≤ 0.05 between means that do not share the letters for each variable in each column.

** planted at a distance: D1= 20 × 60 cm, D2= 40 × 60 cm, D3= 60 × 60 cm.

*** fertilized with NPK fertilizer: F1= N100 P100 K50, F2= N200 P200 K100, F3= N300 P300 K150.

Table S4. Effect of planting distance and NPK fertilization on micronutrient content of *M. stenopetala* leaves.

| treatments | Fe (ppm) | Mn (ppm) | Zn (ppm) |
| --- | --- | --- | --- |
| D1F1 | 308.00 ± 1.50 c | 83.50 ± 1.10 b | 32.60 ± 0.802 c |
| D1F2 | 310.00 ± 2.00 b | 84.60 ± 1.10 b | 34.30 ± 0.802 bc |
| D1F3 | 312.00 ± 2.00 bc | 85.70 ± 1.10 ab | 35.20 ± 0.802 bc |
| D2F1 | 313.00 ± 2.58 bc | 89.50 ± 0.67 ab | 36.70 ± 0.842 ab |
| D2F2 | 316.00 ± 2.58 bc | 89.60 ± 0.67 ab | 36.60 ± 0.842 ab |
| D2F3 | 322.00 ± 2.06 b | 90.70 ± 0.67 ab | 37.80 ± 0.842 ab |
| D3F1 | 336.00 ± 3.16 a | 92.60 ± 0.95 ab | 38.60 ± 0.693 a |
| D3F2 | 338.00 ± 3.16 a | 93.60 ± 0.95 ab | 38.80 ± 0.693 a |
| D3F3 | 340.00 ± 3.16 a | 94.50 ± 0.95 a | 38.80 ± 0.693 a |

* The Tukey test indicates that there is a significant difference at p≤ 0.05 between means that do not share the letters for each variable in each column.

** planted at a distance: D1= 20 × 60 cm, D2= 40 × 60 cm, D3= 60 × 60 cm.

*** fertilized with NPK fertilizer: F1= N100 P100 K50, F2= N200 P200 K100, F3= N300 P300 K150.

Table S5. Effect of planting distance and NPK fertilization on active ingredients of *M. stenopetala* leaves.

| treatments | chlorophyll | total flavonoids | total carotenoids | total tannins | L-ascorbic acid |
| --- | --- | --- | --- | --- | --- |
| D1F1 | 8.42 ± 0.33 b | 1.25 ± 0.036 b | 9.99 ± 0.201 c | 3.02 ± 0.31 c | 230.00 ± 1.15 c |
| D1F2 | 8.33 ± 0.33 b | 1.30 ± 0.036 ab | 11.20 ± 0.201 b | 3.12 ± 0.39 c | 231.00 ± 1.15 c |
| D1F3 | 9.22 ± 0.33 a | 1.32 ± 0.036 ab | 12.60 ± 0.201 a | 3.23 ± 0.39 c | 232.00 ± 1.15 c |
| D2F1 | 7.22 ± 0.34 c | 1.35 ± 0.035 ab | 10.10 ± 0.414 c | 5.28 ± 0.35 b | 240.00 ± 1.53 b |
| D2F2 | 8.22 ± 0.34 b | 1.38 ± 0.035 ab | 10.20 ± 0.514 c | 5.15 ± 0.36 b | 243.00 ± 1.53 b |
| D2F3 | 9.31 ± 0.34 a | 1.42 ± 0.035 ab | 12.80 ± 0.514 a | 5.22 ± 0.36 b | 244.00 ± 1.53 b |
| D3F1 | 8.33 ± 0.36 b | 1.45 ± 0.029 a | 10.30 ± 0.201 c | 6.23 ± 0.35 a | 255.00 ± 1.53 a |
| D3F2 | 8.34 ± 0.36 b | 1.45 ± 0.029 a | 11.60 ± 0.201 b | 6.34 ± 0.36 a | 256.00 ± 1.53 a |
| D3F3 | 9.32 ± 0.36 a | 1.50 ± 0.029 a | 12.92 ± 0.201 a | 6.50 ± 0.36 a | 258.00 ± 1.53a |

* The Tukey test indicates that there is a significant difference at p≤ 0.05 between means that do not share the letters for each variable in each column.

** planted at a distance: D1= 20 × 60 cm, D2= 40 × 60 cm, D3= 60 × 60 cm.

*** fertilized with NPK fertilizer: F1= N100 P100 K50, F2= N200 P200 K100, F3= N300 P300 K150.
